# Imidazoline I_2_ receptor inhibitor idazoxan regulates the progression of hepatic fibrosis via Akt-Nrf2-Smad2/3 signaling pathway

**DOI:** 10.18632/oncotarget.15472

**Published:** 2017-02-18

**Authors:** Li Xuanfei, Chen Hao, Yi Zhujun, Liu Yanming, Gong Jianping

**Affiliations:** ^1^ Department of Hepatobiliary Surgery, The Second Affiliated Hospital of Chongqing Medical University, Chongqing 400010, P. R. China; ^2^ Department of Gastroenterology, The Central Hospital of Wuhan, Tongji Medical College, Huazhong University of Science and Technology, Wuhan 430041, Hubei, P. R. China

**Keywords:** hepatic fibrosis, hepatic stellate cell, imidazoline I_2_ receptor, idazoxan, NF-E2-related factor 2

## Abstract

Liver fibrosis is a global health problem and its relationship with imidazoline I_2_ receptor has not been reported. This study aimed to investigate the effects and underlying mechanisms of imidazoline I_2_ receptor (I_2_R) inhibitor idazoxan (IDA) on carbon tetrachloride (CCl_4_)-induced liver fibrosis. *In vivo* liver fibrosis in mice was induced by intraperitoneally injections of CCl_4_ for eight weeks, and *in vitro* studies were performed on activated LX2 cells treated with transforming growth factor-β (TGF-β). Our results showed that IDA significantly improved liver inflammation, ameliorated hepatic stellate cells activation and reduced collagen accumulation by suppressing the pro-fibrogenic signaling of TGF-β/Smad. Further investigation showed that IDA significantly balanced oxidative stress through improving the expressions and activities of anti-oxidant and detoxifying enzymes and activating Nrf2-the key defender against oxidative stress with anti-fibrotic potentials. Even more impressively, knock out of Nrf2 or suppression of Akt by perifosine (PE) eliminated the anti-oxidant and anti-fibrotic effects of IDA *in vivo* and *in vitro*, suggesting that Akt/Nrf2 constitutes a critical component of IDA's protective functions. Taken together, IDA exhibits potent effects against liver fibrosis via Akt-Nrf2-Smad2/3 signaling pathway, which suggests that specifically targeting I_2_R may be a potentially useful therapeutic strategy for liver fibrosis.

## INTRODUCTION

Liver fibrosis and cirrhosis occur as a result of liver disease including alcoholic liver disease, non-alcoholic steatohepatitis, hepatitis B and C, and autoimmune hepatitis [1–3]. Without appropriate treatment, liver fibrosis eventually leads to cirrhosis in which the normal structure of the functional units in liver becomes disrupted, consequently leading to portal hypertension or hepatic cellular carcinoma (HCC), even ultimately liver failure [4]. Despite some significant progresses in understanding the mechanisms underlying liver fibrosis, current therapies for this disease are still unsatisfactory.

The pathological process of liver fibrosis is characterized by accumulation of excessive extracellular matrix (ECM) proteins [5]. The main component of ECM is a collagen-type IV and laminin-rich compound in normal liver, while switched to a collagen-type I and fibronectin 1 (FN-1)-rich compound in fibrotic liver [3]. It is well accepted that the hepatic stellate cells (HSCs) are the major ECM-producing cells [6]. In response to liver damage, the quiescent HSCs are activated and differentiated into fibrogenic myofibroblast-like cells, which express many ECM proteins including collagen type-I, α-smooth muscle actin (α-SMA), transforming growth factor-β (TGF-β), matrix metalloproteinase (MMP), and tissue inhibitors of metalloproteinase, which contributes to liver fibrosis [7]. Among these proteins, TGF-β is considered to be the most important one which mediates HSC trans-differentiation through the canonical TGF-β/Smad signaling pathway. Upon TGF-β stimulation, regulatory Smads (Smad2, Smad3 and R-Smads) are recruited to TGF-β receptor and activated through phosphorylation. Subsequently, the p-Smad2/3 form complexes with Smad4 and translocate to the nucleus to regulate the transcription of down-stream pro-fibrogenic genes [8].

Over the past decades, much effort has been focused on the role of oxidative stress in fibrogenesis [9, 10]. Oxidative stress derived from hepatocyte injury can induce the elevation of TGF-β level [11]. TGF-β further increases ROS production, whereas generated ROS in turn stimulates TGF-β-associated fibroblast activation and HSCs differentiation [12]. Previous study has shown that oxidative stress is negatively regulated by NF-E2-related factor 2 (Nrf2) [13]. In resting condition, Nrf2 combines with its inhibitor Keap1. Upon ROS or electrophilics stimulation, Nrf2 is disconnected from Keap1 and activated in nuclear as a transcription factor which leads to the transcriptional expression of its downstream antioxidant and detoxifying enzymes, such as hemeoxygenase 1 (HO-1), NAD(P)H: quinoneoxidoreductase 1 (NQO-1), superoxide dismutase (SOD) and catalase (CAT) [14]. What's more, Nrf2 not only balances oxidative stress, but also negatively affects TGF-β-mediated pro-fibrogenic signaling [15, 16], which suggests that Nrf2 could be a potential target for anti-liver fibrosis strategies.

In 1984, the concept of imidazoline receptors was first put forward by Bousquet [17]. Based on the differences of biological characters and body distribution, the imidazoline receptors were divided into three subunits: imidazoline I_1_ receptor (I_1_R), imidazoline I_2_ receptor (I_2_R) and imidazoline I_3_ receptor (I_3_R). I_2_R is thought to be involved in the pathogenesis of many diseases, such as Huntington's disease, Parkinson's disease, type-2 diabetes and glial tumors [18–21]. Several lines of evidence have suggested that inhibition of I_2_R expresses multiple pharmaceutical potentials including suppressing tumor, anti-diabetes and cytoprotection [18, 22]. Our previous studies showed that inhibition of I_2_R reduces LPS-induced oxidative stress and ROS production in macrophages [23]. However, to date, the potential role of I_2_R inhibition in treatment of liver fibrosis has not been reported.

Idazoxan (IDA), a widely used inhibitor for I_2_R, was found to have varied pharmacological effects. Oral administration of IDA could improve the clinical symptoms of patients with bipolar depression, Parkinson's disease or upper respiratory tract illnesses [24–26]. IDA was also found to attenuate blood-brain barrier damage through down-regulating tight junction proteins (JAM-1, Occludin, Claudin-5 and ZO-1) during experimental autoimmune encephalomyelitis in mice [27]. What's more, IDA could suppress production of the inflammatory mediators, such as inducible nitric oxide synthase (iNOS), which may induce hepatocyte damage and participate in development of liver fibrosis [28, 29]. Based on these reports, we proposed that IDA may have protective effects against liver fibrosis.

In this study, we investigated the effects of I_2_R inhibitor IDA on liver fibrosis *in vivo* and *in vitro*. Our results showed that IDA reduced CCl_4_-induced oxidative stress, inhibited TGF-β-associated pro-fibrogenic signaling and attenuated liver fibrosis through its regulation of Akt/Nrf2/Smad signaling pathway. Therefore, this study revealed the protective function and mechanism of IDA, which may indicate inhibition of I_2_R as a potential approach to prevention and treatment of patients with live fibrosis.

## RESULTS

### IDA attenuates the progression of liver fibrosis in CCl_4_-treated mice

**Figure 1:**
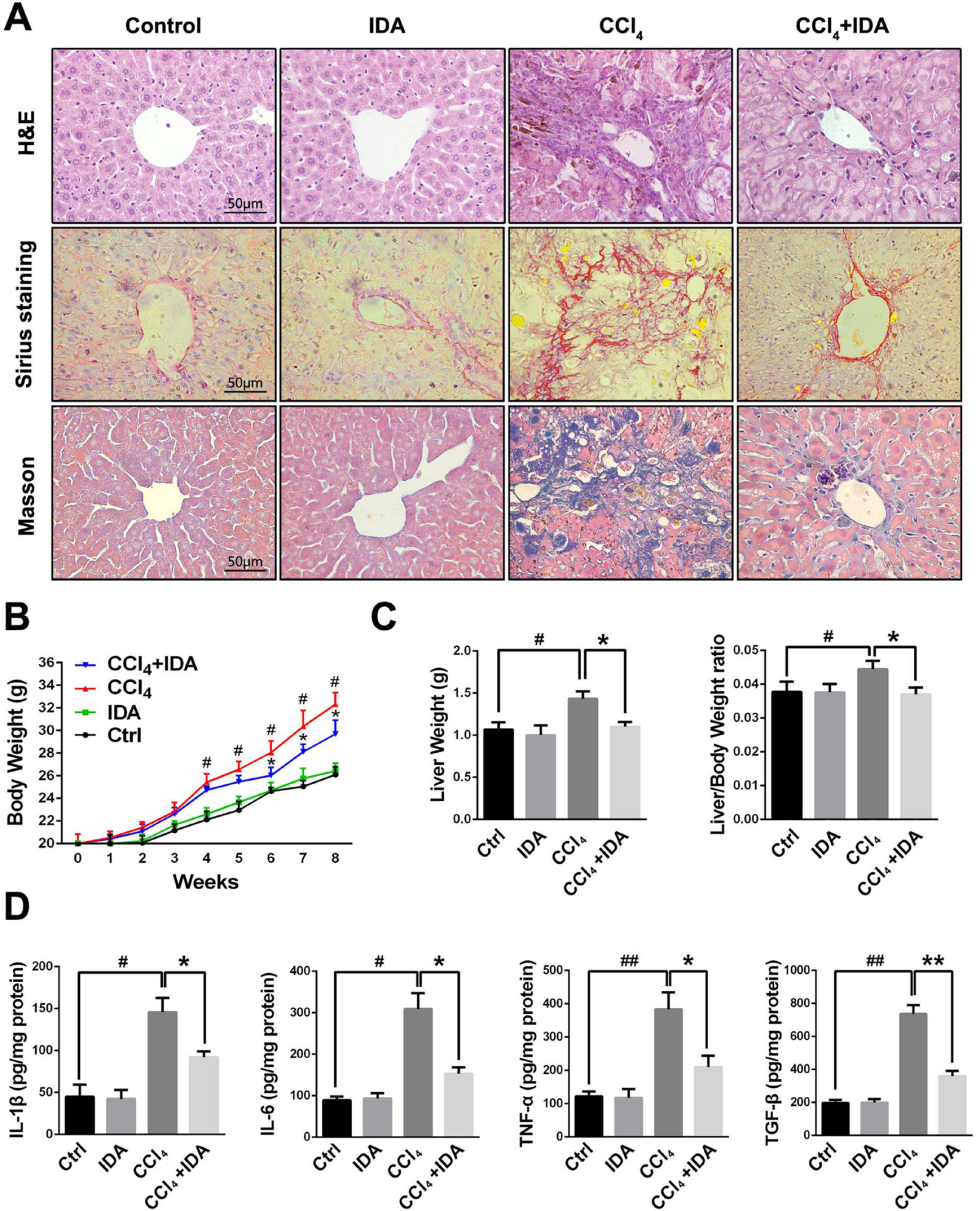
IDA attenuates the progression of liver fibrosis in CCl_4_-treated mice (**A**) Liver fibrosis as assessed by hematoxylin and erosin (H&E), Sirius red Staining and Masson trichrome staining (*n* = 6 in each group). Fibrosis is represented by collagen deposition (red color area) in Sirius Staining and (blue color area) in Masson's trichrome staining. (**B**) Trends in the body weights of C57BL/6 mice were monitored at 1 week intervals throughout the 8 weeks of CCl_4_ treatment. (**C**) The liver weights and liver/body weight ratio at 8 weeks were measured. (**D**) The protein levels of IL-1β, IL-6, TNF-α and TGF-β in liver tissue homogenates were measured by ELISA. The experiments were repeated for three times and data are represented as mean ± SEM. ^#^*p <* 0.05 versus Control; ^##^*p <* 0.01 versus Control; *p < 0.05 versus CCl_4_; **p < 0.01 versus CCl_4_.

As showed in Figure 1A, the liver sections form CCl_4_-treated group showed severe hemorrhagic necrosis, destruction of liver architecture, inflammatory cell infiltration and obvious hepatic ECM deposition. However, IDA treatment remarkably reduced these histopathological changes. To further explore the effects of IDA on CCl_4_-induced liver fibrosis mice, we examined the levels of liver enzymes in serum. The results showed that CCl_4_-induced a significant increase in serum ALT, AST, total bilirubin (TB), total cholesterol (TC) and triglycerides (TG) and a remarkable decrease in serum albumin (Alb), as compared to control group. Treatment with IDA showed a significant improvement in all these values (Table 1). The body weight was significantly increased from injection 4 weeks to injection 8 weeks in CCl_4_ group, while treatment with IDA remarkably decreased the body weight from injection 6 weeks to injection 8 weeks (Figure 1B). Besides, the liver weight and liver/body weight ratio were significantly increased in CCl_4_ group, while they were nearly normal after treated with IDA (Figure 1C). What's more, the levels of IL-1β, IL-6, TNF-α and TGF-β in liver tissue homogenates were also increased significantly as compared to control group, while these cytokines were remarkably reduced in CCl_4_+IDA group (Figure 1D).

**Table 1:** Effects of IDA on hepatotoxicity indices in CCl_4_-treated mice

Groups	ALT (U/L)	AST (U/L)	Alb (g/dl)	TB (mg/dl)	TC (mg/dl)	TG (mg/dl)
Control	21.4 ± 2.1	78.6 ± 4.2	4.1 ± 0.4	2.1 ± 0.3	26.7 ± 3.3	70.4 ± 6.2
IDA	20.8 ± 3.3	79.3 ± 5.1	4.2 ± 0.3	2.3 ± 0.2	28.1 ± 2.4	71.2 ± 5.8
CCl_4_	68.5 ± 4.1^a^	127.9 ± 7.3^a^	2.8 ± 0.2^a^	4.8 ± 0.4^a^	88.5 ± 6.8^a^	167.3 ± 9.5^a^
CCl_4_ + IDA	48.1 ± 3.0^ab^	89.7 ± 5.6^ab^	3.6 ± 0.3^ab^	3.5 ± 0.3^ab^	48.4 ± 4.3^ab^	85.6 ± 7.8^ab^

ALT, Alanine transaminase; AST, Aspartate transaminase; Alb, albumin; TB, total bilirubin; TC, total cholesterol; TG, triglycerides. ^a^*p <* 0.05 versus Control; ^b^*p <* 0.05 versus CCl_4_. Statistical analyses for two groups comparisons were performed using Student's *t* test. Statistical analysis for multiple group comparisons was performed using one-way analysis of variance (ANOVA) followed by Duncan's test.

### IDA inhibits TGF-β signaling in the liver of CCl_4_-induced mouse and TGF-β-treated LX2 cells

**Figure 2:**
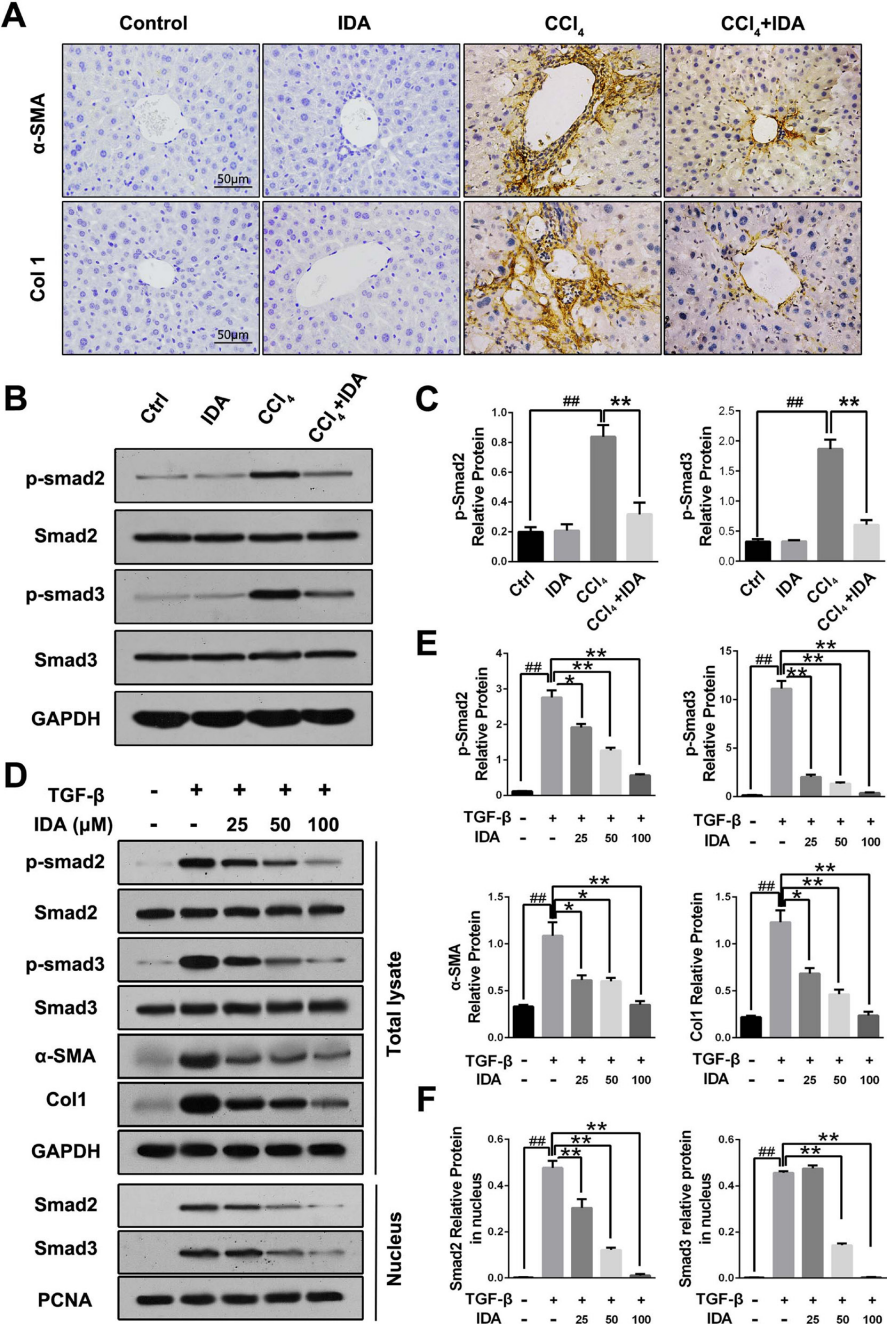
IDA inhibits TGF-β/Smad signaling in CCl_4_-treated mice and TGF-β-treated LX2 cells (**A**) The expression levels of α-SMA and Col1 in liver tissue were determined by immunohistochemistry. (**B**) The expression of p-Smad2, p-Smad3, Smad2 and Smad3 in liver tissue were measured by western blotting. (**C**) Quantification of Figure 2B were shown. ^##^*p <* 0.01 versus Control; **p < 0.01 versus CCl_4_. (**D**) LX2 cells were pretreated with series doses of IDA (25 μM, 50 μM or 100 μM) for 1 h and then treated with TGF-β (5 ng/ml) for 1 h. Then p-Smad2, Smad2, p-Smad3 and Smad3 in total lysate and Smad2 and Smad3 in nucleus were detected by western blotting. (**E**–**F**) Quantification of Figure 2D. The experiments were repeated for three times and data are represented as mean ± SEM. ^##^*p <* 0.01 versus Control; *p < 0.05 versus TGF-β; **p < 0.01 versus TGF-β.

As shown in Figure 2A, 2D, Supplementary Figures 1 and 2, both CCl_4_ treatment in mice and TGF-β stimulation in LX2 cells induced a significant increases in the expressions of α-SMA and Col1, while treatment with IDA remarkably reduced the expressions of α-SMA and Col1. TGF-β/Smad signaling is the major pathway leading to the excessive ECM accumulation and essential for the progression of liver fibrosis. To further explore the mechanism underlying the anti-fibrotic effect of IDA, we examined the Smad signaling in the liver of CCl_4_-induced mice and TGF-β-treated LX2 cells. The results showed that the phosphorylation and nuclear translocation of Smad2 and Smad3 were significantly increased *in vitro* and *in vivo*, however, treatment with IDA significantly reduced the phosphorylation and nuclear translocation of Smad2 and Smad3 (Figure 2B–2F).

### IDA balances oxidative stress and improves the expression and activity of anti-oxidant and detoxifying enzymes

**Figure 3:**
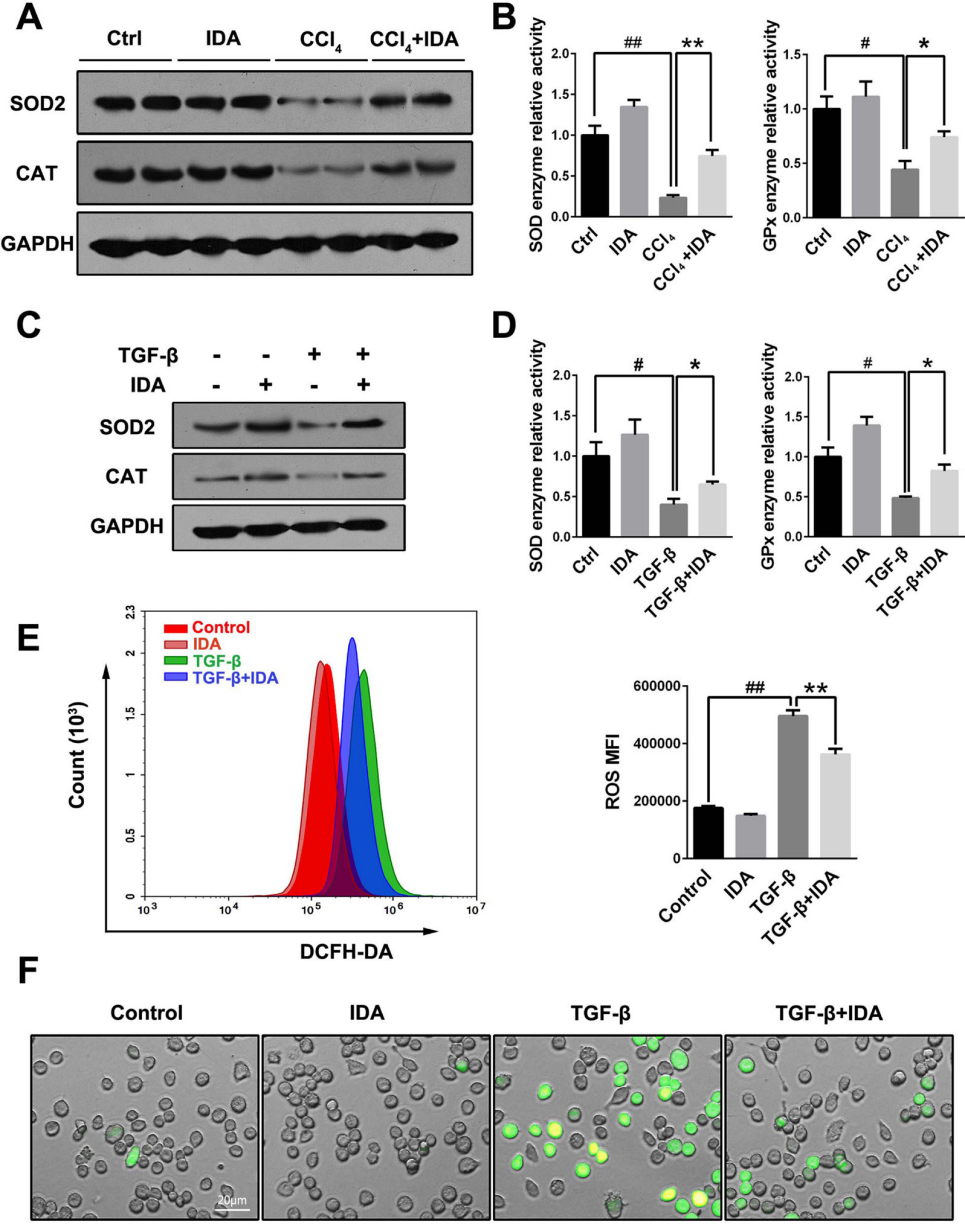
IDA balances oxidative stress and increases the expressions and activities of antioxidant and detoxifying enzymes (**A**) The expressions of superoxide dismutase 2 (SOD2) and catalase (CAT) in liver were measured by western blotting (Two randomly selected samples were presented). (**B**) The relative enzyme activities of SOD and Glutathione Peroxidase (GPx) in liver were measured by enzyme activity detection kits. (**C**) LX2 cells were pretreated with IDA (100 uM) for 1 h and then treated with TGF-β (5 ng/ml) for 12 h. The expression of SOD2 and CAT were detected by western blotting. (**D**) The relative enzyme activities of SOD and GPx in LX2 cells were measured. (**E**) Intracellular ROS assay. LX2 cells were pretreated with IDA (100 uM) for 1 h and then treated with or without TGF-β (5 ng/ml) for 18 h. Intracellular ROS were measured by flow cytometry (DCFH-DA). The mean fluorescent intensity of intracellular ROS (ROS MFI) were shown. (**F**) The images of ROS were shown with a fluorescent microscope. The experiments were repeated for three times and data are represented as mean ± SEM. Statistical analyses for two groups comparisons were performed using Student's *t* test. Statistical analysis for multiple group comparisons was performed using one-way analysis of variance (ANOVA) followed by Duncan's test. ^#^*p <* 0.05 versus Control; ^##^*p <* 0.01 versus Control; *p < 0.05 versus CCl_4_ or TGF-β; **p < 0.01 versus CCl_4_ or TGF-β.

Oxidative stress has been shown to play an important role in the progression of liver fibrosis. As shown in Figure 3A–3D, both CCl_4_ treatment in mice and TGF-β stimulation in LX2 cells induced a significant reduce in the expressions of SOD2 and CAT and enzyme activities of SOD and GPx, while treatment with IDA remarkably increased expressions and activities of these enzymes (Figure 3A–3D). To test whether IDA could affect TGF-β-induced oxidative stress, we used DCFH-DA to detect the intracellular reactive oxidant species (ROS). The results showed that TGF-β significantly increased the intracellular ROS productions in LX2 cells, while treatment with IDA remarkably reduced the ROS productions (Figure 3E, 3F).

### IDA activates Nrf2 signaling in CCl_4_-treated mice and TGF-β-treated LX2 cells

**Figure 4:**
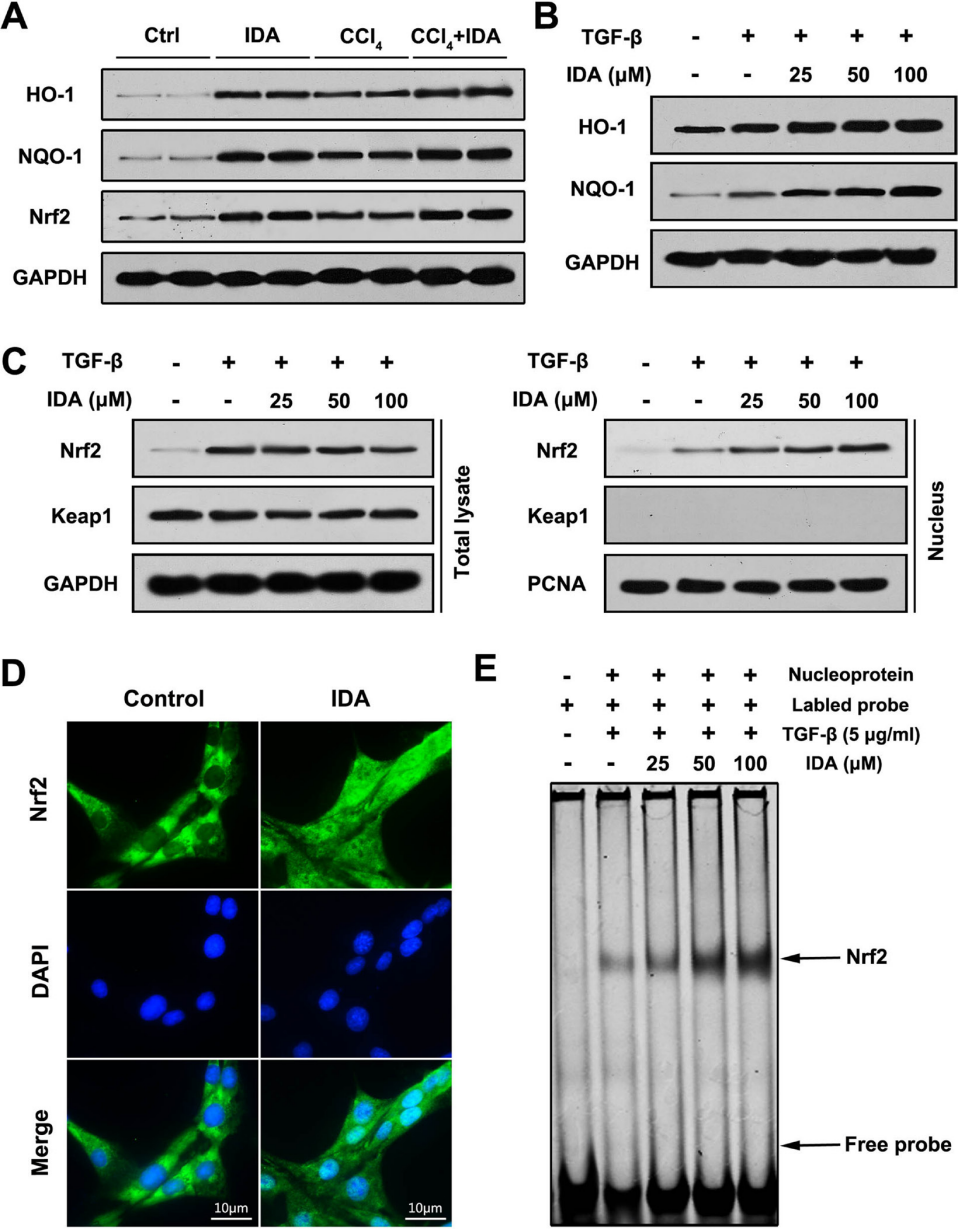
IDA activates Nrf2 signaling in CCl4-treated mice and TGF-β-treated LX2 cells (**A**) The expressions of HO-1, NQO-1 and Nrf2 in liver were determined by western blotting (Two randomly samples in each group were presented). (**B**–**C**) LX2 cells were pretreated with series doses of IDA (25 uM, 50 uM or 100 uM) for 1 h and then treated with or without TGF-β (5 ng/ml) for 1 h. The expressions of HO-1 and NQO-1 were assayed by western blotting. The expressions of Nrf2 and keap1 in total lysate and nucleus were measured by western blotting. (**D**) LX2 cells were treated with PBS or IDA (100 uM) for 1 h. The nuclear translocations of Nrf2 were determined by immunofluorescence assay. (**E**) LX2 cells were pretreated with series doses of IDA (25 uM, 50 uM or 100 uM) for 1 h and then treated with or without TGF-β (5 ng/ml) for 1 h. The DNA binding activity of Nrf2 was measured by EMSA.

To identify the upstream events that mediate IDA anti-oxidative stress function, we focused on the Nrf2 signaling pathway. We first examined the expression of Nrf2 and its downstream proteins (HO-1 and NQO-1) *in vivo*. The results showed that the protein levels of HO-1, NQO-1 and Nrf2 were significantly higher in CCl_4_ group than control group, while treatment with IDA displayed additional increments (Figure 4A). As showed in Figure 4B, IDA enhanced TGF-β induced protein expressions of HO-1 and NQO-1 in a dose-dependent manner *in vivo*. The nuclear translocation of Nrf2 is an essential step for Nrf2 activation. Treated with TGF-β increased the nuclear expression of Nrf2, but had no effect on the expression of Keap1 in LX2 cells (Figure 4C). IDA promoted the nuclear translocation of Nrf2 in a dose-dependent manner (Figure 4C and 4D). What's more, as showed in Figure 4E, IDA increased the DNA binding activity of Nrf2 in a dose-dependent manner. Besides, we detected the effects of IDA on p38 MAPK signaling. The results showed that p38 MAPK were also activated in CCl_4_-treated mice, however, IDA has little effects on p38 phosphorylation *in vivo* and *in vitro* (Supplementary Figure 3).

### IDA improves oxidative stress, Smad signaling and liver fibrosis in a Nrf2 dependent pathway

**Figure 5:**
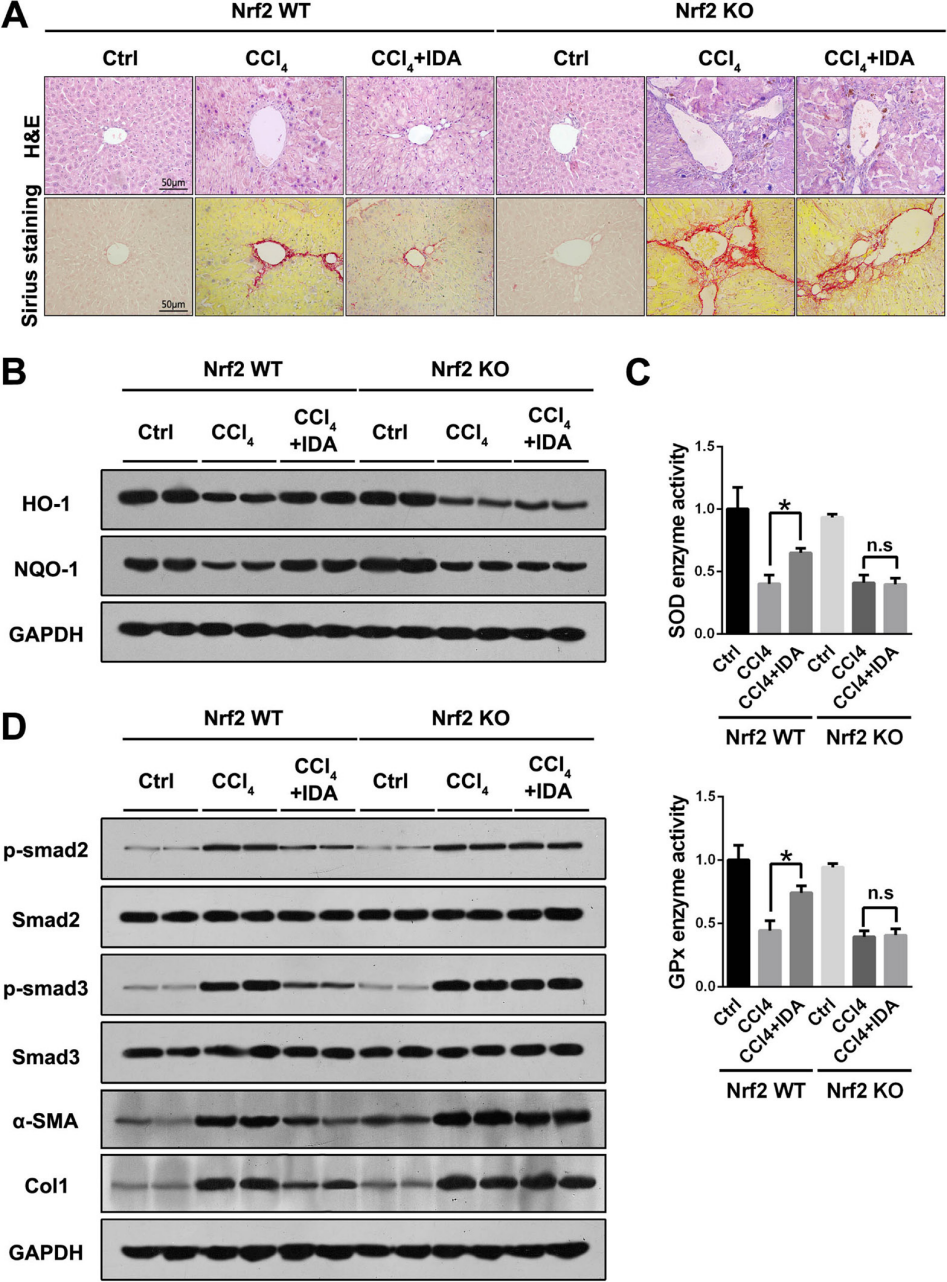
IDA inhibits oxidative stress, Smad signaling and liver fibrosis in a Nrf2 dependent pathway (**A**) Representative images of H&E and Sirius red staining of liver sections from Nrf2 WT or Nrf2 KO mice were shown. (**B**) The expressions of HO-1 and NQO-1 in liver were detected by western blotting (Two random selected samples in each group). (**C**) The relative enzyme activities of SOD and GPx in liver tissues were measured. (**D**) The expressions of p-Smad2, Smad2, p-Smad3, Smad3, α-SMA and Col1 in liver tissues were measured by western blotting. (Two random selected samples in each group). *p < 0.05 vs CCl_4_; n.s. = No significant. Statistical analyses were performed using Student's *t* test.

To further investigate whether the anti-oxidant and anti-fibrotic effect of IDA was dependent on the Nrf2 signaling pathway, we examined the effects of IDA in Nrf2 knockout mice. As shown in Figure 5A, IDA significantly improved the liver damage and fibrosis in Nrf2 WT mice, however, its protective effects were abrogated in Nrf2 KO mice (Figure 5A). Furthermore, IDA increased the expression of HO-1 and NQO-1, reversed the low relative enzyme activities of SOD and GPx, inhibited the phosphorylation of Smad2 and Smad3 and decreased the levels of α-SMA and Col1, but failed to do so in Nrf2 KO mice (Figure 5B–5D).

### Knockdown of Nrf2 blocks the inhibitory effects of IDA on TGF-β/Smad signaling in LX2 cells

**Figure 6:**
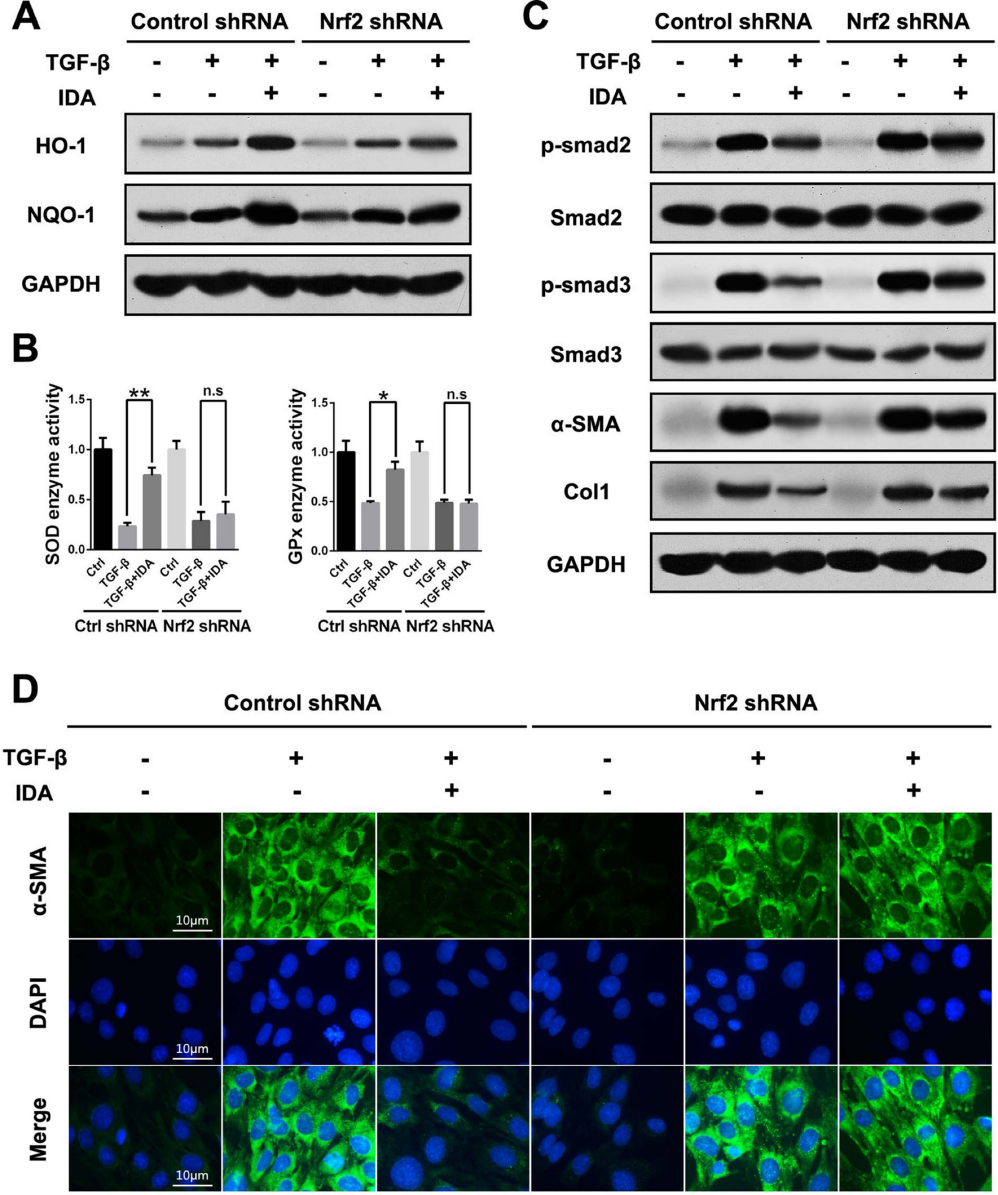
Knockdown of Nrf2 blocks the inhibitory effects of IDA on TGF-β/Smad signaling in LX2 cells LX2 cells were transfected with control shRNA or Nrf2 shRNA Lentivirus Particles, then treated with IDA (100 μM) for 1 h and next treated with or without TGF-β (5 ng/ml) for 12 h. (**A**) The expressions of HO-1 and NQO-1 were detected by western blotting. (**B**) The relative enzyme activity of SOD and GPx were measured by ELISA. (**C**) The expressions of p-Smad2, Smad2, p-Smad3, Smad3, α-SMA and Col1 were detected by western blotting. (**D**) The expression of α-SMA was measured by immunofluorescence assay. Data are represented as mean ± SEM. *p < 0.05 vs TGF-β; n.s. = No significant. Statistical analyses were performed using Student's *t* test.

Then we investigated the effects of IDA in TGF-β-treated LX2 cells after transfection with control shRNA or Nrf2 shRNA. The results showed that IDA increased the expression of HO-1 and NQO-1, improved the enzyme activities of SOD and GPx, and inhibited the levels of p-Smad2, p-Smad3, α-SMA and Col1 in control shRNA group, while all these effects of IDA were abolished in Nrf2 shRNA group (Figure 6A–6D). Thus, we concluded that IDA inhibited TGF-β/Smad signaling in LX2 cells via the activation of Nrf2 pathway.

### P-Akt is essential for the anti-fibrotic effects of IDA in CCl_4_-treated mice and TGF-β-treated LX2 cells

**Figure 7:**
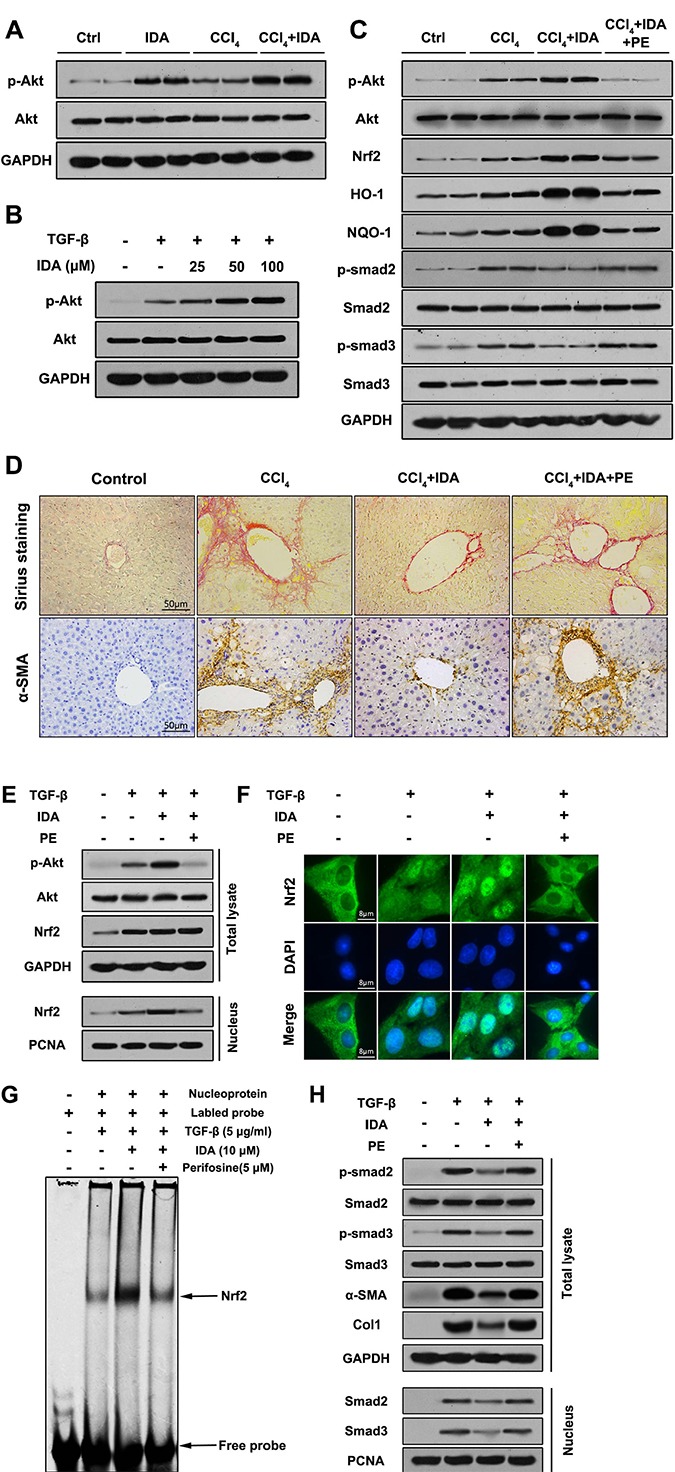
P-Akt is essential for the anti-fibrotic effects of IDA in CCl_4_-treated mice and TGF-β-treated LX2 cells (**A**) The expressions of p-Akt and Akt in liver tissues were measured by western blotting (Two randomly samples in each group were represented). (**B**) LX2 cells were pretreated with series doses of IDA (25 μM, 50 μM or 100 μM) for 1 h and then treated with or without TGF-β (5 ng/ml) for another 1 h. The expressions of p-Akt and Akt were measured by western blotting. (**C**) C57BL/6 mice were injected with CCl_4_, IDA (20 mg/kg/wk) and (or) Perifosine (60 mg/kg/wk) for 8 weeks. The p-Akt, Akt, Nrf2, HO-1, NQO-1, p-Smad2, Smad2, p- Smad3 and Smad3 in liver tissues were measured by western blotting (Two random selected samples in each group). (**D**) Representative images of Sirius Red staining and immunohistochemistry of α-SMA in liver. (*n* = 6 in each group). (**E**) LX2 cells pretreated with PE (5 μM) and (or) IDA (100 μM) for 1 h and next treated with or without TGF-β for 1 h. The p-Akt, Akt and Nrf2 in total lysate and Nrf2 in nucleus were measured by western blotting. (**F**) The nuclear translocations of Nrf2 were determined by immunofluorescence assay. (**G**) The DNA binding activity of Nrf2 were measured by EMSA. (**H**) The p-Smad2, p-Smad3, Smad2, Smad3, α-SMA and Col1 in total lysates and Smad2 and Smad3 in nucleus were measured by western blotting.

PI3K-Akt was considered to be one of the upstream pathways of Nrf2-HO-1/NQO-1 signaling. Therefore, we sought to investigate whether IDA induces Nrf2 activation via PI3K-Akt signaling. In our study, the results showed that Akt signaling was activated in the liver of CCl_4_-treated mice and TGF-β-treated LX2 cells. Interestingly, treatment with IDA significantly enhanced the expression of p-Akt *in vivo* and *in vitro* (Figure 7A–7B). Then we use perifosine (PE), a Akt inhibitor, to suppress the phosphorylation of Akt (Figure 7C, 7E). The results showed that PE abolished the effect of IDA on Nrf2-HO-1/NQO-1 and TGF-β**/**Smad signaling (Figure 7C, 7E, 7H). Besides, PE blocked the effect of IDA on the nuclear translocation and DNA binding activity of Nrf2 (Figure 7F, 7G). Finally, PE eliminated the inhibitory effect of IDA on ECM accumulation in the liver of CCl_4_-induced mice and TGF-β-treated LX2 cells (Figure 7D, 7H). Thus, we concluded that IDA regulated Nrf2/HO-1/TGF-β/Smad signaling pathway by phosphorylation of Akt.

## DISCUSSION

Liver fibrosis is the common pathological hallmark of chronic liver injury which causes excessive ECM accumulation in liver. Recently, it has been evidenced that even advanced liver fibrosis is still reversible [30, 31]. Thus, it is critically important to develop novel and effective methods to reverse liver fibrosis. In this study, IDA, an I_2_R inhibitor, displayed significant effects against liver fibrosis as evidenced by the improved liver enzyme levels and the attenuation of histopathological changes. Moreover, IDA was effective in suppressing the Akt-Nrf2-Smad2/3 signaling pathway to inhibit HSCs activation and attenuate the progression of hepatic fibrosis *in vivo* and *in vitro*.

TGF-β is the most important pro-fibrogenic cytokine. Activation of TGF-β/Smad signaling pathway plays an important role in liver fibrosis through accumulation of ECM, especially collagen type-I and secretion of fibrogenic cytokines in HSCs [32]. It has been largely demonstrated that disruption of TGF-β/Smad signaling shows a protective effect against tissue fibrosis. Tang et al. reported that asiatic acid inhibited liver fibrosis by up-regulation of Smad7 and thus blocking the activation of TGF-β/Smad2/3 signaling pathway [33]. HYDAMTIQ, a selective PARP-1 inhibitor, down-regulated the expression of TGF-β and p-Smad3, and thus exhibited an anti-fibrotic effect in bleomycin-induced lung fibrosis [34]. In this study, IDA exhibited a potent anti-fibrotic effect by decreasing the production inflammatory cytokines, reducing the expression of α-SMA and Col1, ameliorating the pathological changes, and inhibiting the phosphorylation and the nuclear translocation of Smad2 and Smad3 *in vivo* and *in vitro*.

Oxidative stress has been reported to play a predominant pro-fibrogenic role in liver fibrosis [35]. Liver fibrosis-associated oxidative stress is due to increased ROS and decreased antioxidant capacity. Nrf2 is an important transcription factor that plays an essential role in protecting against oxidative stress. Many products such as sulforaphane [16, 36], dimethylfumarate [15], oleanolic [37], isorhamnetin [38] and sinomenine [39] have been reported to attenuate tissue fibrosis via activation of Nrf2 signaling pathway, which suggests a causal relationship between Nrf2 and TGF-β/Smad signaling pathway. In our study, we found that IDA significantly increased expression of Nrf2 and its downstream proteins in mice and LX2 cells. Then we used Nrf2 KO mice and Nrf2 knockdown LX2 cells to determine whether IDA inhibits TGF-β/Smad via Nrf2 signaling pathway. The results showed that the anti-fibrotic effect of IDA was eliminated in Nrf2 KO mice and Nrf2 knockdown cells, which demonstrated that IDA exhibited an anti-fibrotic effect via Nrf2 signaling pathway.

Some evidences have also reported that mitogen-activated protein kinase 14 (p38 MAPK) signaling is involved in the ROS production and liver fibrosis [40–42]. Peng et al. showed that activation of Nrf2 by dioscin could inhibit p38 MAPK phosphorylation and thus reduced the degree of liver fibrosis [43]. In this study, we found that p38 MAPK signaling is also activated in CCl_4_-treated mice, however, IDA has little effects on p38 phosphorylation. This might be because IDA has a different anti-fibrotic molecular mechanisms with dioscin.

PI3K/Akt is a multifunctional signaling pathway which plays key roles in the regulation of cell apoptosis, proliferation and differentiation [44]. In mammalian cells, PI3K/Akt is an important upstream regulator of the Nrf2 and its activation can lead to Nrf2 nuclear translocation [45, 46]. Crosstalk between PI3K/Akt and Nrf2 signaling pathway has been largely investigated. Some products such as agmatine, punicalagin, s-propargyl-cysteine and salvianolic acid B up-regulated the Nrf2 expression via the activation of PI3K/Akt pathway [23, 47–49]. In this study, we found that IDA induced a significant increase of p-Akt while the total Akt levels remained unchanged *in vivo* and *in vitro*. Then, we used perifosine (PE), a PI3K/Akt inhibitor, to further investigate whether IDA inhibits Nrf2/Smad signaling through Akt. The results showed that PE not only reduced the phosphorylation of Akt, but also abolished the effect of IDA on Nrf2/Smad signaling, and finally eliminated the protect effects of IDA on CCl_4_-induced liver fibrosis. *In vitro* experiment, we also found that PE diminished the effects of IDA on Nrf2/Smad signaling. Therefore, we concluded that IDA enhanced the nuclear translocation of Nrf2 and inhibited TGF-β/Smad signaling pathway by phosphorylation of Akt.

**Figure 8:**
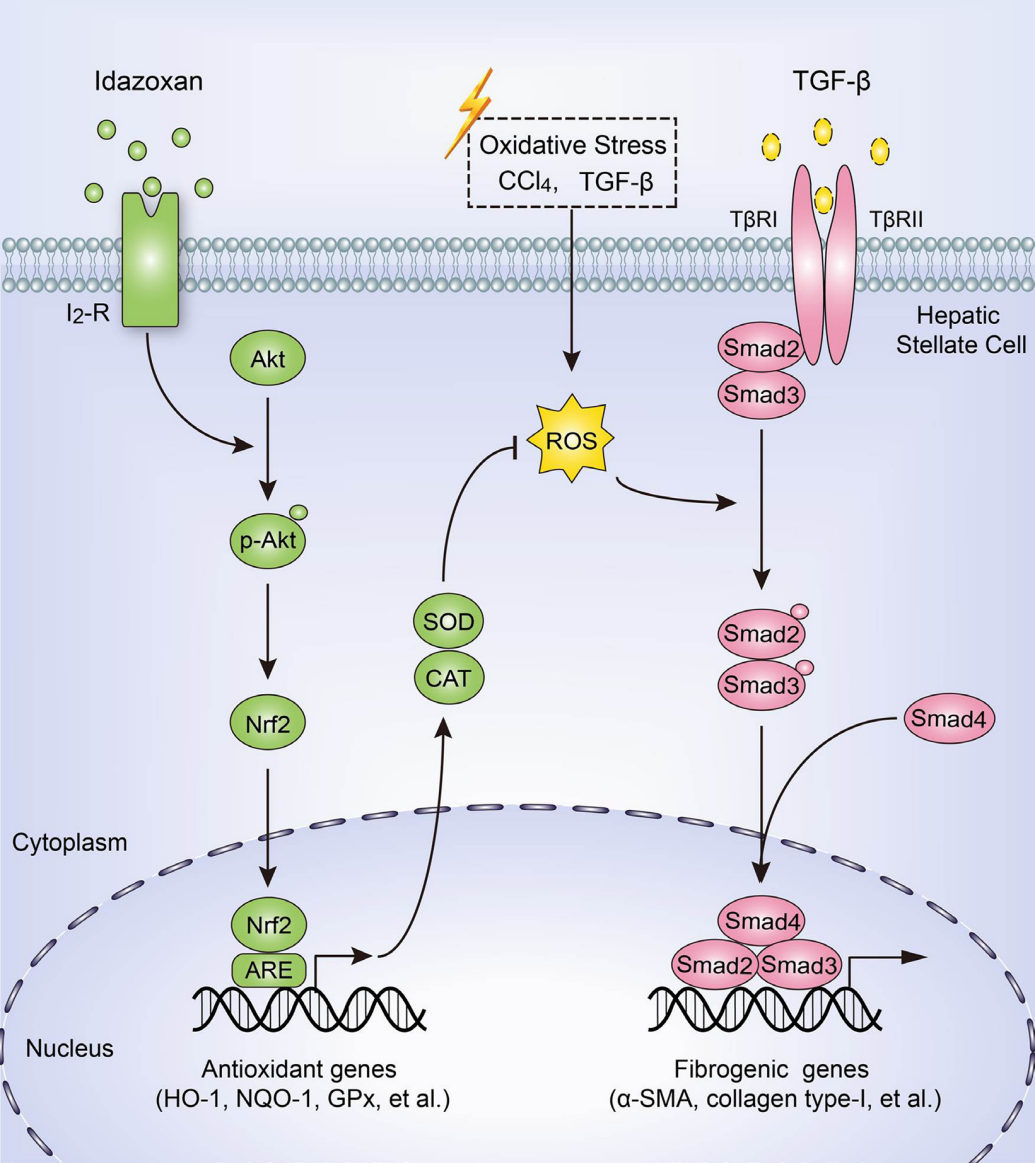
Schematic diagram of the anti-fibrotic effect of idazoxan in inhibition of HSCs activation Idazoxan induced Nrf2 nuclear translocation by increasing the phosphorylation of Akt, leading to the expression of antioxidant proteins, thereby blocked ROS-induced Smad2/3 signaling, resulting in the inhibition of HSC activation.

In conclusion, we demonstrate that IDA effectively balances oxidative stress, inhibits ECM expression and ameliorates liver fibrogenesis by Akt-Nrf2-Smad2/3 signaling pathway (Figure 8). These findings provide strong evidence supporting I_2_R inhibitor idazoxan as an anti-hepatic fibrosis medicine. To date, the relationship between liver fibrosis and I_2_R has not been reported. Our findings may provide potential applications of I_2_R inhibition in treatment of liver fibrosis in the future.

## MATERIALS AND METHODS

### Reagents

Idazoxan (IDA) and transforming growth factor-β (TGF-β) were purchased from Sigma-Aldrich (St. Louis, MO, USA). Carbon tetrachloride (CCl_4_) was purchased from Xilong Chemistry Plant (Shantou, China). Perifosine (PE), anti-Nrf2, anti-HO-1, anti-α-SMA and anti-Col1 were purchased from Abcam (Cambridge, UK). Anti-NQO1, anti-Smad2, anti-phospho-Smad2, anti-Smad3, anti-p-Smad3, anti-keap1, anti-GAPDH, anti-PCNA, anti-SOD2, anti-Catalase, anti-p38, anti-p-p38, anti-Akt and anti-p-Akt were obtained from Santa Cruz (CA, USA). IL-1β, IL-6, TNF-α and TGF-β enzyme-linked immunosorbent assay (ELISA) kits were obtained from Boster Biotechnology (Wuhan, China). Reactive Oxygen Species Assay Kit, BCA Protein Assay Kit, Nuclear and Cytoplasmic Extraction kits, Total Superoxide Dismutases Assay Kit, glutathione perosidase (GPx) Assay Kit and Catalase Assay Kit were purchased from Beyotime Biotechnology (Shanghai, China).

### Animals

C57BL/6 mice weighing 19–23g were obtained from the Chongqing Medical University Animal Center. Nrf2^–/–^ (Nrf2 KO; Nfe^2l2tm1Ywk^/J) mice and Nrf2+/+ (Nrf2 WT; C57BL/6J) mice were purchased from Jackson Laboratory (No. 017009; Bar Harbor, Maine, USA). Mice were housed in a climate-controlled, circadian rhythm-adjusted room and were under a 12-h dark/light cycle with free access to food and water. This study was strictly carried out according to the recommendations in the Guide for the Care and Use of Laboratory Animals of the National Institutes of Health (NIH Publication, 8th edition, 2011). The protocol was approved by the committee on the Ethics of Animal Experiments of Chongqing Medical University.

After 1 week of acclimatisation, mice were randomly divided into four groups (*n* = 8 per group) as follows: control group, IDA group, CCl_4_ (fibrosis) group, and CCl_4_+IDA (treatment of fibrosis) group. Eight mice in CCl_4_ group or CCl_4_+IDA group received intra-peritoneal injection of 10% CCl_4_ dissolved in olive oil (0.02 ml/g) biweekly. Eight mice in IDA group or CCl_4_+IDA group were treated with IDA at a dose of 3 mg/kg body weight biweekly. Eight control mice received an isovolumetric dose of olive oil as that of the CCl_4_. The body weight of mice were measured every week. At 8 week, the mice were sacrificed. The weight of livers were measured, and the liver tissues were harvested and stored at -80°C for further experiments.

### Cell culture and transfection

Human hepatic stellate cell line-LX2 cell was purchased from MEIXUAN Biotechnology Company (Shanghai, China) and cultured at 37°C in a normoxic atmosphere containing 5% CO_2_ with high-glucose Dulbecco's modified Eagle's medium (Gibco, Thermo Fisher Scientific, MA, USA) supplemented with 10% fetal bovine serum (FBS, Gibco, Thermo Fisher Scientific, MA, USA), 100 U/ml of penicillin, and 100 mg/ml of streptomycin. The LX2 cells were transfected with control shRNA and Nrf2 shRNA Lentivirus (Santa Cruz, CA, USA) according to the manufacturer's protocol.

### Cell proliferation assay

The LX2 cells were seeded into 96-well plates at the density of 5 × 10^4^ cells/mL and incubated for 24 h, and then treated with different concentrations of IDA (6, 12.5, 25, 50, 100, 200, 400, 800, or 1600 μM) for 24 h. Cell proliferation was measured by MTT method.

### Histological analysis

The liver tissues were fixed in 4% buffered paraformaldehyde overnight and then embedded in paraffin. The sections were cut at 6 um thick and stained with H&E, Masson trichrome and Sirius red staining by standard procedures, respectively.

### Enzyme-linked immunosorbent assay (ELISA)

The concentrations of IL-1β, IL-6 and TNF-α in liver tissue homogenates were measured by mouse ELISA kits (Wuhan Boster Biological Technology Co., Ltd.). The procedures were in accordance with manufacturers’ instructions strictly. The absorbance was measured at 450 nm.

### Immunohistochemistry

α-SMA and Col1 staining were performed using the PV-9002 Polink-2 plus^®^ Polymer HRP Detection System (ZSGB-BIO, Beijing, China) according to manufacturer's instructions. After antigen retrieval, α-SMA monoclonal antibody was diluted 1:100 in goat serum and Col1monoclonal antibody was diluted 1:200 in goat serum. After staining, slides were counterstained using a standard haematoxylin and ethanol dehydration protocol. The positive areas were observed and captured by microscope.

### Western blotting

Aliquots of 50 ug per sample of cell lysate, liver tissue homogenates or nucleus fractionation were electrophoresed on 10% SDS-PAGE gel, and proteins were transferred to a PVDF (polyvinylidene fluoride) membrane. The membranes were kept with blocking buffer, followed by incubating with primary antibody at 4°C overnight and then incubated with appropriate secondary antibody conjugated to horseradish peroxidase (HRP) for 1 h. The protein expression was detected by enhanced chemiluminescence (ECL) reagent. The blot intensity was quantified by the Image J software. All western results were normalized to GAPDH or PCNA.

### Measurement of antioxidant enzyme activities

Intracellular ROS in LX2 cells were detected by dichloro-dihydro-fluorescein diacetae (DCFH-DA). LX2 cells were treated with IDA in presence or absence of TGF-β for 18 h, then cells were incubated with DCFH-DA (10 uM) supplemented with DMEM for 30 min and washed with phosphate-buffered saline (PBS) for three times. Intracellular ROS were measured by flow cytometry at 530 nm channel. The fluorescent images were captured with a fluorescence microscope.

The enzymatic activities of GPx and SOD were measured by enzyme activity detection kits (Beyotime, Shanghai, China) according to manufacturers’ instructions. The reaction of GPx was incubated at 25°C and the absorbance was measured at 340 nm every 4 min. The reaction of SOD was incubated at 37°C for 30 min and the absorbance was determined at 450 nm.

### Immunofluorescence assay

The LX2 cells were seeded into 12-well plates (2 × 10^5^ cells/well) with aseptic cover lips. After incubation, the cells were washed with PBS, fixed with 4% paraformaldehyde for 10 min and permeabilised with 0.5% Triton-X in PBS for 10 min at room temperature. The cells were incubated with 5% bovine serum albumin (BSA) to block the nonspecific binding sites. A primary antibody against Nrf2 (1:100 dilution) was incubated with the fixed cells at 4°C overnight. Then the cells were incubated with fluorescence-labelled secondary antibody for 1 h. Finally, DAPI was used to stain the nuclear of cells for 10 min. And the images were captured by a fluorescence microscope.

### Electrophoretic mobility shift assay (EMSA)

EMSA was performed as described previously [23]. Briefly, nuclear proteins from treated LX2 cells were extracted and then quantified by BCA assay. To measure the binding capacity of the nuclear proteins with Nrf2 binding domain (ARE), the DNA-protein complexes were resolved on 6.5% mini Gels for 40 min at 180V and detected at 700 nm using the Odyssey scan bed (LiCor, Lincoln, NE, USA). The probe consisted of an oligonucleotide containing the ARE 5′-TTT TATGCTGTGTCATGGTT-3′.

### Statistical analysis

All experiments were repeated for three times and data are represented as mean ± SEM. An independent sample *t-test* was used to determine the statistical differences between two groups. Statistical analysis for multiple group comparisons was performed using one-way analysis of variance (ANOVA) followed by Duncan's test. All tests were conducted with SPSS 17.0 software (SPSS Inc., Chicago, USA). The results with a *p <* 0.05 was considered statistically significant and *p <* 0.01 was considered highly significant.

## SUPPLEMENTARY MATERIALS FIGURES AND TABLES


