# Supplementary material for: Imidazoline I2 receptor inhibitor idazoxan regulates the progression of hepatic fibrosis via Akt-Nrf2-Smad2/3 signaling pathway
Source: Oncotarget. 2017 Feb 18;8(13):21015–30. doi: 10.18632/oncotarget.15472 (PMC5400562; doi:10.18632/oncotarget.15472)
Supplement: Supplementary file 1 [file oncotarget-08-21015-s001.pdf]

Imidazoline I² receptor inhibitor idazoxan regulates the progression of hepatic fibrosis via Akt-Nrf2-Smad2/3 signaling pathway

Supplementary Materials

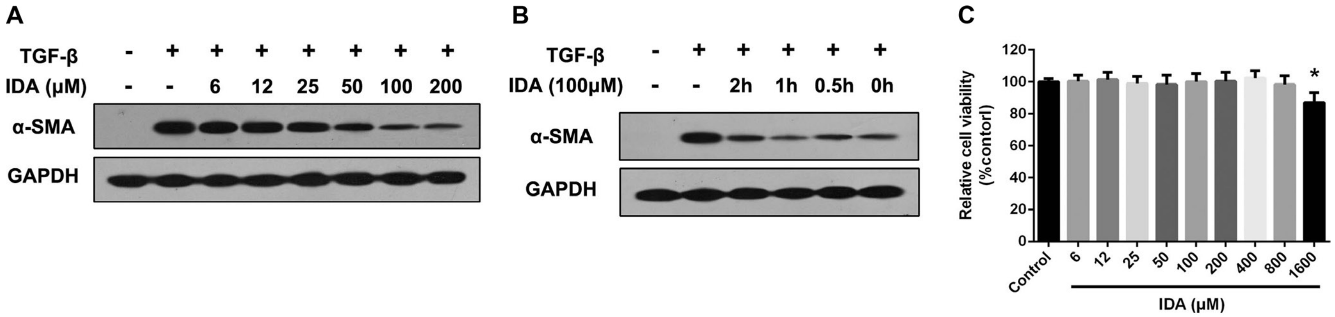

Supplementary Figure 1: Effects of IDA on the activation of LX2 cells. (**A**) LX2 cells were pretreated with series doses of IDA (6 μM, 12.5 μM, 25 μM, 50 μM, 100 μM or 200 μM) for 1h and then treated with TGF-β (5 ng/ml) for 12 h. The expression of α-SMA were detected by western blotting. (**B**) LX2 cells were pretreated with IDA (100 μM) for 2 h, 1 h, 0.5 h or 0h and then treated with TGF-β (5 ng/ml) for 12 h. The expression of α-SMA were detected by western blotting. (**C**) LX2 cells were treated with series doses of IDA (6 μM, 12.5 μM, 25 μM, 50 μM, 100 μM, 200 μM, 400 μM, 800 μM, or 1600 μM) for 24 h. The cell viability were detected by MTT assay. The experiments were repeated for three times and data are represented as mean ± SEM. *$p < 0.05$ versus control.

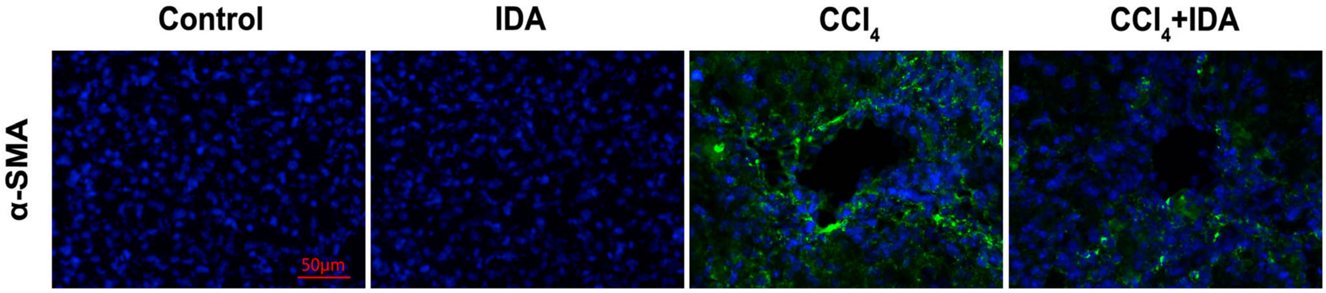

Supplementary Figure 2: IDA inhibits α-SMA expression in CCl4-treated mice. The expression levels of α-SMA in liver tissue were determined by immunofluorescence.

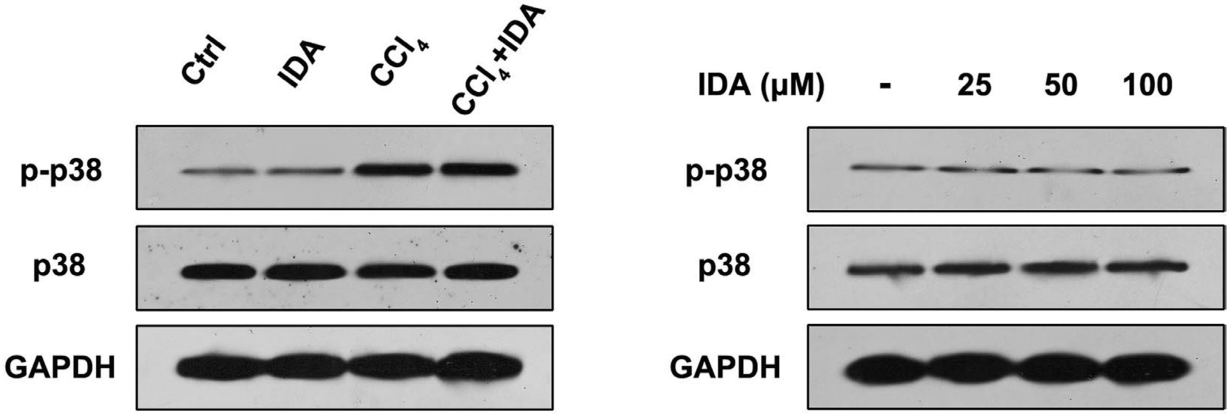

Supplementary Figure 3: Effects of IDA on p38 MAPK signaling. (**A**) The expressions of p-p38 and p38 in liver tissues were measured by western blotting. (**B**) LX2 cells were pretreated with series doses of IDA (25 μM, 50 μM or 100 μM) for 1 h. The expressions of p-p38 and p38 were measured by western blotting.
